# Efficacy of Intermittent or Continuous Very Low-Energy Diets in Overweight and Obese Individuals with Type 2 Diabetes Mellitus: A Systematic Review and Meta-Analyses

**DOI:** 10.1155/2020/4851671

**Published:** 2020-01-27

**Authors:** Yi Shan Huang, Qiyan Zheng, Huisheng Yang, Xinwen Fu, Xueqin Zhang, Chenhui Xia, Zebing Zhu, Yu Ning Liu, Wei Jing Liu

**Affiliations:** ^1^Renal Research Institution of Beijing University of Chinese Medicine, Dongzhimen Hospital Affiliated to Beijing University of Chinese Medicine, Beijing 100700, China; ^2^Institute of Acupuncture and Moxibustion, China Academy of Chinese Medical Sciences, Beijing 100700, China; ^3^Department of Endocrinology Nephropathy, Dongzhimen Hospital Affiliated to Beijing University of Chinese Medicine, Beijing 100700, China; ^4^Institute of Nephrology, Zhanjiang Key Laboratory of Prevention and Management of Chronic Kidney Disease, Guangdong Medical University, Zhanjiang, Guangdong 524001, China

## Abstract

**Objective:**

This study is aimed at investigating the efficacy of a very low-energy diet (VLED) in overweight and obese individuals with type 2 diabetes mellitus (T2DM).

**Methods:**

We thoroughly searched eight electronic resource databases of controlled studies concerning the efficacy and acceptability of intermittent or continuous VLEDs in patients with T2DM compared with other energy restriction interventions.

**Results:**

Eighteen studies (11 randomized and seven nonrandomized controlled trials) with 911 participants were included. The meta-analyses showed that compared with a low-energy diet (LED) and mild energy restriction (MER), VLED is superior in the reduction of body weight (mean difference (MD) MD_LED_ = −2.77, 95% confidence interval (CI) CI_LED_ = −4.81 to − 0.72, *P*_LED_ = 0.008; MD_MER_ = −6.72, 95%CI_MER_ = −10.05 to − 3.39, *P*_LED_ = 0.008; MD_MER_ = −6.72, 95%CI_MER_ = −10.05 to − 3.39, *P*_LED_ = 0.008; MD_MER_ = −6.72, 95%CI_MER_ = −10.05 to − 3.39, *P*_LED_ = 0.008; MD_MER_ = −6.72, 95%CI_MER_ = −10.05 to − 3.39, *P*_LED_ = 0.008; MD_MER_ = −6.72, 95%CI_MER_ = −10.05 to − 3.39, *P*_LED_ = 0.008; MD_MER_ = −6.72, 95%CI_MER_ = −10.05 to − 3.39, *P*_LED_ = 0.008; MD_MER_ = −6.72, 95%CI_MER_ = −10.05 to − 3.39, *I*^2^ = 0%) and TG level (MD = −0.25, 95%CI = −0.55 to 0.06, *P*_LED_ = 0.008; MD_MER_ = −6.72, 95%CI_MER_ = −10.05 to − 3.39, *I*^2^ = 0%) and TG level (MD = −0.25, 95%CI = −0.55 to 0.06, *P*_LED_ = 0.008; MD_MER_ = −6.72, 95%CI_MER_ = −10.05 to − 3.39, *P*_LED_ = 0.008; MD_MER_ = −6.72, 95%CI_MER_ = −10.05 to − 3.39, *P*_LED_ = 0.008; MD_MER_ = −6.72, 95%CI_MER_ = −10.05 to − 3.39, *P*_LED_ = 0.008; MD_MER_ = −6.72, 95%CI_MER_ = −10.05 to − 3.39, *P*_LED_ = 0.008; MD_MER_ = −6.72, 95%CI_MER_ = −10.05 to − 3.39,

**Conclusion:**

Dietary intervention through VLEDs is an effective therapy for rapid weight loss, glycemic control, and improved lipid metabolism in overweight and obese individuals with T2DM. Thus, VLEDs should be encouraged in overweight and obese individuals with T2DM who urgently need weight loss and are unsuitable or unwilling to undergo surgery. As all outcome indicators have low or extremely low quality after GRADE evaluation, further clinical trials that focus on the remission effect of VLEDs on T2DM are needed.

## 1. Introduction

It is well known that obesity is a major risk factor for type 2 diabetes mellitus (T2DM) [1] and the majority of patients with T2DM are overweight or obese [2]. Obesity management is confirmed as an effective strategy in the prevention and remission of T2DM [3].

Multiple strategies including diet, physical activity, behavioural therapy, pharmacologic therapy, and bariatric surgery are recommended for obesity management [3]. In previous evidence-based clinical guidelines, dietary modification is recommended as a fundamental aspect of diabetes care, based on its benefits on glycemia and HbA1c levels [4]. Recently, several studies suggest that short-term and more extreme dietary energy restriction aiming on intensive weight loss can even reverse some cases of T2DM [5–7]. Very low-energy diet (VLED) has been confirmed as an effective and safe option for weight loss in obese individuals [8]. There is no standard definition of a VLED programme across different countries and continents [9–11]. However, a VLED is generally defined as a very low total energy intake (≤800 kcal/day) [8, 10]. Recently, a growing body of studies focus on the efficacy and acceptability of VLEDs in patients with T2DM who are overweight or obese [12–14] and propose that VLEDs may be an underutilized therapy for patients with T2DM. Intermittent VLED is an alternative strategy of continuous VLEDs for T2DM, which typically involves periods of VLEDs interchanged by periods of *ad libitum* energy intake or mild energy restriction (MER, a slight diet intervention method which provides energy less than *ad libitum* energy intake but more than 1600 kcal/day) [15, 16]. The efficacy of both intermittent and continuous VLED should be considered.

A low-energy diet (LED) containing 800–1600 kcal/day is also considered an option of clinical obesity management of patients with T2DM [17, 18], but the difference in efficacy and safety between VLEDs and LEDs is rarely discussed. Bariatric surgery is recommended for obese patients (body mass index (BMI), 35.0–39.9 kg/m^2^) with T2DM who did not achieve durable weight loss and improvement in comorbidities with reasonable surgical methods [3]. For example, Roux-en-Y gastric bypass (RYGB), as currently one of the most effective types of bariatric surgery, achieves energy limitation by reducing stomach capacity and reducing dietary intake. However, bariatric surgeries have more adverse effects and complications compared with energy restriction strategy. Moreover, VLEDs may produce a similar effect on glycemic control, *β*-cell function, and insulin sensitivity as bariatric surgeries. Thus, it is necessary to evaluate the efficacy of VLEDs compared with other methods of energy restriction in overweight and obese individuals with T2DM.

A previous systematic review among overweight and obesity individuals with T2DM found that VLED has benefits of weight loss and glycemic control [19]. However, the systematic review included a small number of participants, and the long-term effect of VLEDs is unclear. Another recently published systematic review found that VLED programmes in children and adolescents with obesity induce short- to medium-term weight loss and also demonstrated significant improvements in diabetic outcomes, such as HbA1c and glucose levels [10]. Recently, several clinical studies have been conducted to compare VLEDs with other energy restriction methods. Thus, it is necessary to investigate the efficacy of VLEDs in overweight and obese adult individuals with T2DM. Our systematic review and meta-analyses are aimed at clarifying the effect of VLEDs on weight loss, glycemic control, and blood lipid levels in overweight and obese individuals with T2DM and further exploring the long-term efficacy of VLEDs to provide more substantial evidence in the clinical application of VLEDs.

## 2. Materials and Methods

### 2.1. Search Strategy

We comprehensively searched PubMed, EMBASE, Cochrane Library, Web of Science, SINOMED, China National Knowledge Infrastructure, WanFang, and Chongqing VIP Information databases from inception until July 2019 for clinical trials investigating intermittent or continuous VLEDs for overweight and obese adults with T2DM. Additional studies were searched in the reference lists of all identified publications, including relevant meta-analyses and systematic reviews.

### 2.2. Inclusion Criteria

Published and unpublished randomized controlled trials (RCTs) and non-RCTs, which are clinical controlled studies evaluating the efficacy of intermittent or continuous VLEDs and qualitative studies exploring the acceptability of, barriers to, and facilitators of VLEDs, were considered for inclusion in this review.

We included clinical studies that satisfied the following criteria: (1) participants in the included studies were overweight or obese (mean BMI ≥ 30 kg/m^2^ or ≥10% above the ideal body weight based on the Metropolitan Life Insurance Company's tables); (2) adults (aged ≥18 years) had T2DM in older studies using a different measure of obesity; (3) studies used intermittent or continuous VLEDs comprising ≤800 kcal/day in at least one intervention arm; and (4) studies also had to include a control arm receiving other energy control methods, including LEDs (800-1600 kcal/day), bariatric surgery, and MER. We excluded clinical studies with the following features: (1) both the intervention and comparator arms received VLED treatment (except VLEDs after surgical treatment) and (2) the intervention is VLED combined with other weight loss drugs. If a study compared three or more arms, VLED arms were considered to be the intervention and other energy control methods the comparators.

The outcome indicators of this study include the following: (1) weight loss (kg), (2) fasting plasma glucose levels (mmol/l) and change in medication, (3) triglyceride (TG) level (mmol/l), (4) homeostatic model assessment of insulin resistance (HOMA-IR) level, (5) dropout, (6) side effects, and (7) rebound.

### 2.3. Data Extraction

Two reviewers (YS Huang and XW Fu) independently extracted data from original trial reports using a standardized form. Data extracted included study characteristics (first author, publication year, single center or multicenter, sample size, intervention and control, period of treatment, and follow-up duration), characteristics of patients (inclusion criteria, background treatments, mean age, proportion of men, baseline weight, and baseline glucose levels), reported outcomes (weight, fasting plasma glucose levels, and adverse events), and information on methodology. We contacted the study authors when we needed to obtain additional information that was unavailable in the online publications or supplementary materials.

### 2.4. Quality Assessment

Risk of bias of RCTs was assessed using the Cochrane Collaboration's tool [20]. We evaluated non-RCTs according to the Risk Of Bias In Non-randomised Studies of Interventions (ROBINS-I) tool [21]. Two investigators independently completed the assessments, and discrepancies were discussed with a third party and resolved by consensus.

Additionally, the Grading of Recommendations, Assessment, Development, and Evaluation (GRADE) framework was used to assess the quality of evidence contributing to each network estimate, which characterizes the quality of a body of evidence on the basis of the study limitations, imprecision, inconsistency, indirectness, and publication bias for the primary outcomes [22].

### 2.5. Statistical Analyses

The data entry and analysis were conducted using Microsoft Excel 2016 and Review Manager software version 5.3, respectively. Risk ratio and standard mean difference with 95% confidence interval (CI) of the outcomes were calculated as effect measure. The *I*^2^-statistic was calculated for heterogeneity, as a measure of the proportion of the overall variation that is attributable to between-study heterogeneity. A fixed-effects (FE) model was used if *I*^2^ < 50%; otherwise, the random-effects model was used.

To assess whether the results were influenced by study characteristics (effect modifiers), a subgroup analysis was conducted according to the study duration (<12 or ≥12 months). Additionally, sensitivity analyses were performed before combining RCTs and non-RCTs in the meta-analyses to determine possible additional sources of heterogeneity and changes in effect sizes.

Publication bias was tested by visual inspection of the funnel plots. When few studies are included in the analysis, the power of the tests is too low; therefore, publication bias was only examined if >10 study comparisons were included in the analysis [23].

## 3. Results

### 3.1. Study Characteristics

**Figure 1:**
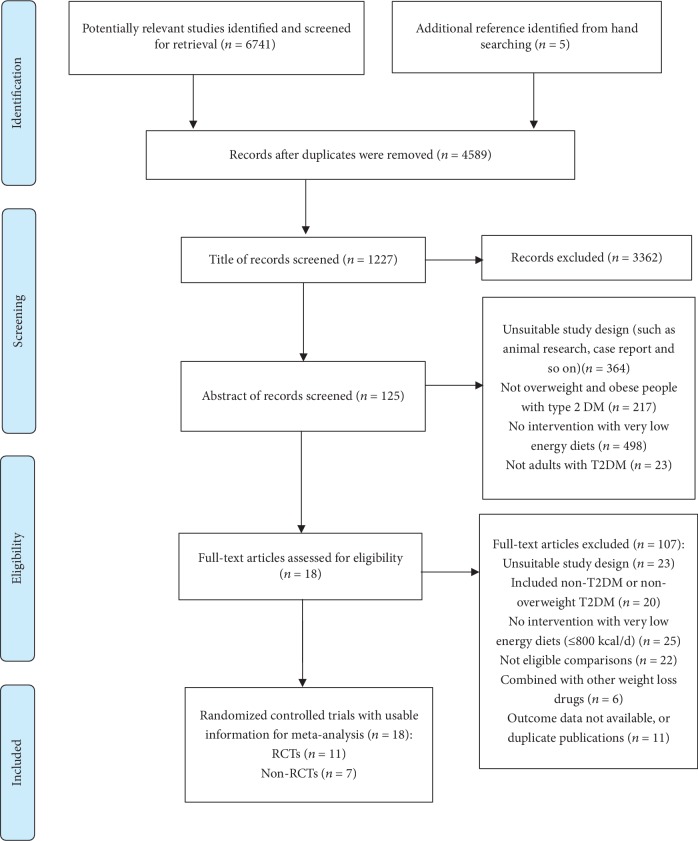
Flow chart of literature search and selection.

The search identified 6746 studies, of which 2157 were duplicates. Then, 4589 titles and abstracts were screened, with 145 studies for full-text screening. Finally, 18 eligible studies (911 participants) [24–41] evaluated the effects of intermittent or continuous VLEDs on overweight or obese patients with T2DM compared with other energy control methods, and specifically, 7 studies (583 participants) [25–31] compared VLEDs with LEDs, 6 studies (204 participants) [36–41] with MER, and 5 studies (124 participants) [24, 32–35] with bariatric surgery. Particularly, among the five studies involving surgical treatment, four studies (Jackness et al. [24], Lips et al. [32], Plum et al. [33], and Steven et al. [35]) used gastric bypass and 1 study (Cinkajzlova et al. [34]) used a variety of surgical approaches, including gastric plication (10 participants), gastric banding (2 participants), and gastric bypass (1 participant). Seven of the 18 included studies were non-RCTs. All of them were observational studies, four of them (Jackness et al. [24], Lips et al. [32], Plum et al. [33], and Cinkajzlova et al. [34]) compared VLEDs with bariatric surgery, and 3 of them (Paisey et al. [36–38]) compared VLEDs with MER.  Figure 1 shows the screening process.  Table 1 shows the main characteristics of included trials.

### 3.2. Evaluation of the Risk of Bias of the Selected Studies

**Figure 2:**
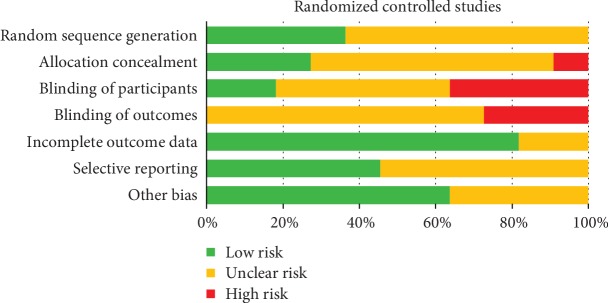
Risk of bias summary of included randomized trials with the Cochrane risk of bias tool.

The risk of bias for the included RCTs was assessed using the Cochrane risk of bias tool. None of the RCTs had an overall low risk of bias. Most RCTs had unclear risk of bias for sequence generation, allocation concealment, blinding of participants, blinding of outcome, and selective reporting because no detailed information was provided. However, three studies had high risk of bias for blinding of participants and blinding of outcome assessment, and one study had high risk of bias for allocation concealment because it could not be performed. Moreover, there is incomplete outcome data that most studies had a low risk of bias. Risk of bias assessment of included trials is shown in Figure 2.

**Figure 3:**
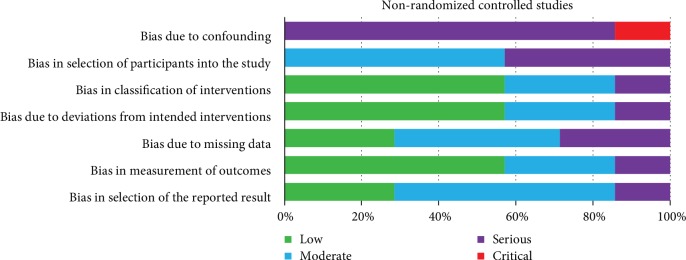
Risk of bias summary of included nonrandomized trials with the ROBINS-I tool.

The risk of bias for the included non-RCTs according to the ROBINS-I tool is presented in Figure 3. None of the studies had a low or moderate risk of bias, six (Jackness et al. [24], Lips et al. [32], Plum et al. [33], and Paisey et al. [36–38]) had signs of serious bias, and one (Cinkajzlova et al. [34]) had critical bias. The domain “bias due to confounding” was a main source of critical or serious risk of bias. The domain “bias in selection of participants into the study” had moderate or serious risk of bias in all studies. Risk of bias assessment is shown in Figure 3.

### 3.3. Meta-Analysis

#### 3.3.1. Weight Loss

**Figure 4:**
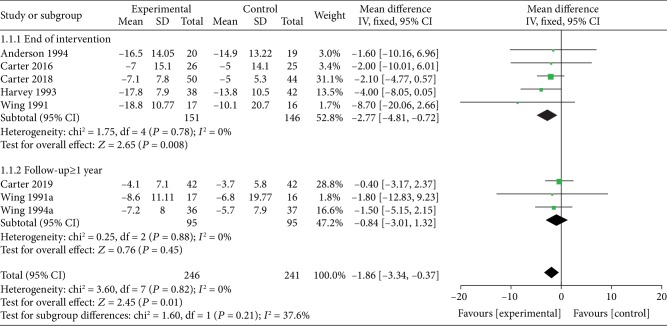
Forest plot on the mean difference in weight loss between VLED and LED controls.

*(1) VLEDs versus LEDs*. Seven studies [25–31] analyzed weight loss when a VLED (*n* = 246) was compared with a LED (*n* = 241). Five of the studies provided data at the end of the intervention, and three provided data in the long-term follow-up (≥1 year). Subgroup analyses did result in differences in various time points. When the intervention is completed, the VLED group lost significantly more weight than the comparator arms (MD = −2.77; 95%CI = −4.81, −0.72; *P* = 0.008, <0.05; *I*^2^ = 0%). However, when follow-up is ≥1 year, the observed difference in weight loss compared with controls was not significant (MD = −0.84; 95%CI = −3.01, 1.32; *P* = 0.45; *I*^2^ = 0%) (Figure 4).

**Figure 5:**
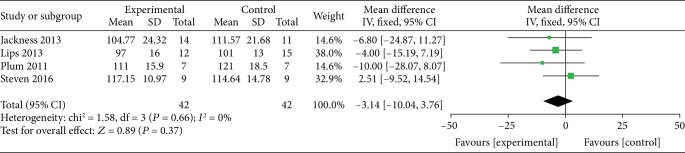
Forest plot on the mean difference in weight loss among VLED and bariatric surgery controls.

*(2) VLEDs versus Bariatric Surgery*. Four studies [24, 32, 33, 35] analyzed the weight loss between the VLED and surgery groups, including 84 participants. Moreover, the surgical methods used in these four studies were RYGB as comparator arms. The merged data with no evidence of interstudy heterogeneity (*I*^2^ = 0%), according to the DerSimonian-Laird FE model, revealed that the VLEDs and RYGB have similar effects on weight loss, and there is no significant difference between them (MD = 2.51; 95%CI = −9.52, 14.54; *P* = 0.37, >0.05) (Figure 5).

**Figure 6:**
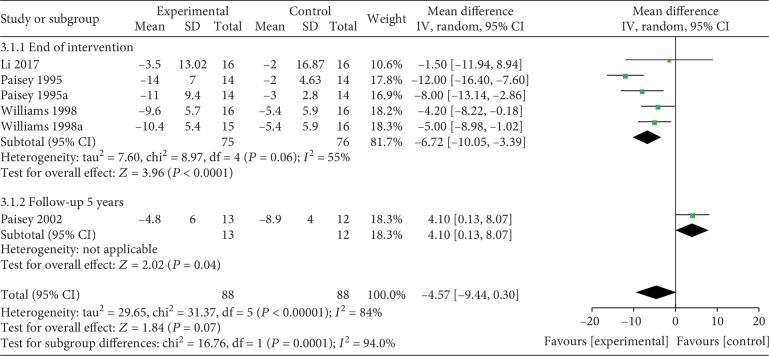
Forest plot on the mean difference in weight loss between VLED and MER controls.

*(3) VLEDs versus MER*. Four studies [37–40] analyzed the weight loss when a VLED (*n* = 88) was compared with MER (*n* = 88). Three studies provided data at the end of the intervention, and one provided data for long-term follow-up (5 years). In particular, the study of Williams et al. [40] contains two types of VLED interventions, and that of Paisey et al. [38] contains data for two endpoints. According to the results of the subgroup analysis, the data at the end of the intervention showed that VLED was significantly better than MER in weight loss (MD = −6.72; 95%CI = −10.05, −3.39; *P* < 0.0001), with evidence of moderate heterogeneity (*I*^2^ = 55%; *P*_heterogeneity_ = 0.06). Sensitivity analysis showed that the heterogeneity was 0% when “Paisey et al. [38]” was removed, and the effect of VLEDs on weight loss was still significantly better than that of the control (MD = −5.19; 95%CI = −7.6, −2.78; *P* < 0.0001). However, when followed up for 5 years, similar to the result of the “Paisey et al. [37]” study, MER was better maintained than VLEDs (MD = 4.1; 95%CI = 0.13, 8.07; *P* = 0.06) (Figure 6).

#### 3.3.2. Blood Glucose and Changes in Medication

**Figure 7:**
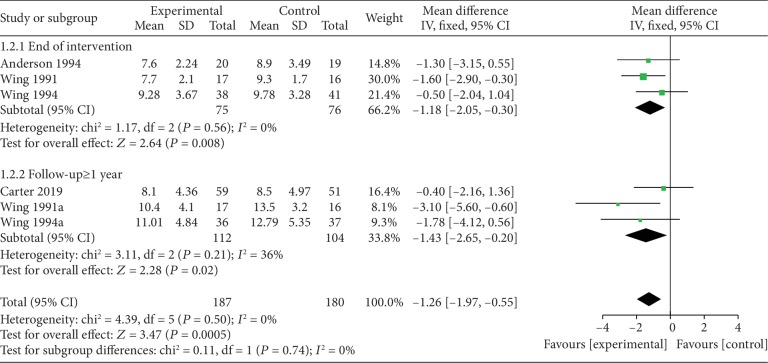
Forest plot on the mean difference in blood glucose levels between VLED and LED controls.

*(1) VLEDs versus LEDs*. Four studies [25, 28, 30, 31] analyzed the blood glucose levels between the VLED and LED groups, and all of them provided data at the end of the intervention. Simultaneously, two provided data for long-term follow-up (≥1 year). A significant difference in weight change in favor of the intervention arm was noted at both the end of the intervention (MD = −1.18; 95%CI = −2.05, −0.30; *P* = 0.008, <0.05) and follow-up (MD = −1.43; 95%CI = −2.65, −0.20; *P* = 0.02, <0.05), and both of them had no evidence of interstudy heterogeneity (*I*^2^ = 0%). Regarding the use of hypoglycemic drugs, Carter et al. reported that although medication dose decreased with time, all participants using medication at baseline were also using medication at the end of the study. At 2 years, one study (Wing et al. [31]) reported that fewer participants in the VLED group required medication (45% vs. 69% in the VLED and LED groups, respectively) (Figure 7).

**Figure 8:**
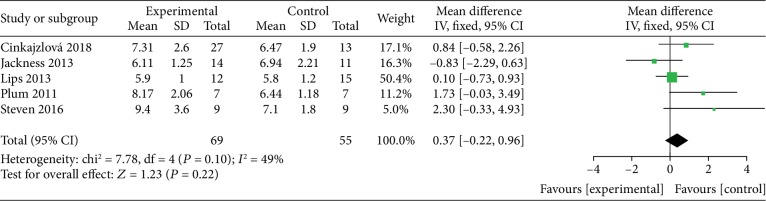
Forest plot on the mean difference of glucose among VLED and bariatric surgery controls.

*(2) VLEDs versus Bariatric Surgery*. Five studies [24, 32–35] analyzed the blood glucose levels between the VLED (*n* = 69) and bariatric surgery groups (*n* = 55). The merged data with no evidence of interstudy heterogeneity (*I*^2^ = 49%), according to the DerSimonian-Laird FE model, revealed that VLEDs and surgery have similar effects on weight loss, and there is no significant difference between them (MD = 0.37; 95%CI = −0.22, 0.96; *P* = 0.22, >0.05) (Figure 8). In the use of hypoglycemic drugs, one study [33] showed that all hypoglycemic drugs were discontinued in the RYGB arm and decreased by 55% in the VLED arm after the intervention. In another study [32], metformin was reintroduced in 4/15 participants in the RYGB arm and 2/12 participants in the VLED arm after the intervention, and the difference was not significant.

**Figure 9:**
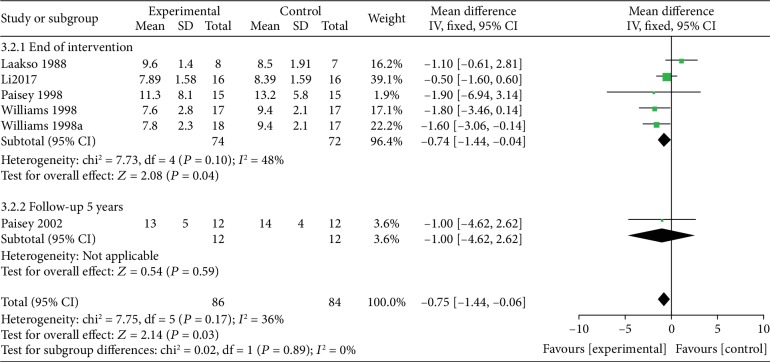
Forest plot on the mean difference in blood glucose levels between VLED and MER controls.

*(3) VLEDs versus MER*. Five studies [36, 37, 39–41] analyzed the blood glucose levels between the VLED (*n* = 86) and MER groups (*n* = 84). Results from the subgroup analyses showed that VLED was significantly better than MER in lowering blood glucose levels (MD = −6.72; 95%CI = −10.05, −3.39; *P* < 0.0001) at the end of the intervention, with evidence of low heterogeneity (*I*^2^ = 48%; *P*_heterogeneity_ = 0.17). However, at the 5-year follow-up, only one study by “Paisey et al. [37]” reported that the difference in blood glucose levels compared with controls was not significant (MD = −1; 95%CI = −4.62, 2.62; *P* = 0.59). In the use of hypoglycemic drugs at the end of the intervention, the study of Paisey et al. showed that, at 6 months (all patients who underwent VLEDs had reverted to normal food for at least two weeks), the patients in the VLED group discontinued insulin, sulphonylureas, or hypolipidemic agents, while patients in the MER group were not able to discontinue their antidiabetic or hypolipidemic therapies. At 1 year, 14 of 15 patients in the VLED group, but none in the conventional diet group, had discontinued insulin and any oral hypoglycemic medication (Figure 9).

#### 3.3.3. TG

**Figure 10:**
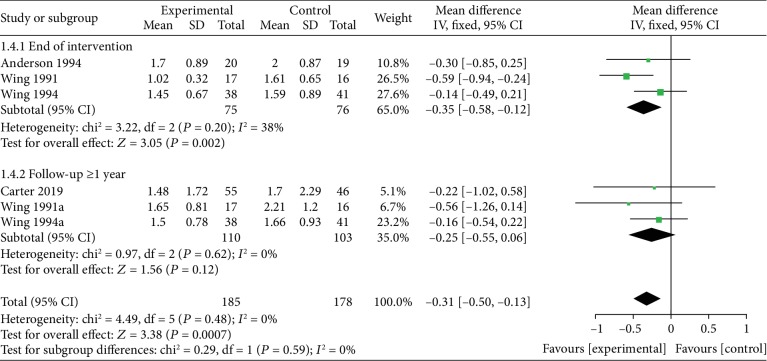
Forest plot on the mean difference in TG levels between VLED and LED controls.

*(1) VLEDs versus LEDs*. Four studies [25, 28, 30, 31] analyzed the TG level between the VLED (*n* = 185) and LED groups (*n* = 179). All studies provided data at the end of the intervention, and two provided data in the long-term follow-up (≥1 year). Results from subgroup analyses showed that the VLED group had significantly lower TG level than the comparator arms at the end of the intervention (MD = −0.35; 95%CI = −0.58, −0.12; *P* = 0.002, <0.05; *I*^2^ = 38%). However, when the follow-up duration is ≥1 year, the observed difference in the TG level compared with controls was not significant (MD = −0.25; 95%CI = −0.55, 0.06; *P* = 0.12, >0.05; *I*^2^ = 0%) (Figure 10).

**Figure 11:**
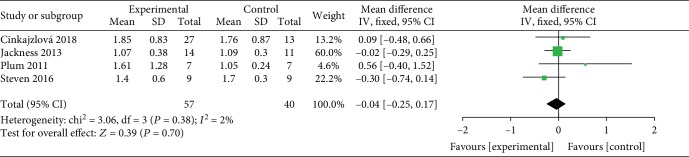
Forest plot on the mean difference in TG levels between VLED and bariatric surgery controls.

*(2) VLEDs versus Bariatric Surgery*. Four studies [24, 33–35] analyzed the TG levels between the VLED (*n* = 57) and bariatric surgery groups (*n* = 40). The merged data, which had no evidence of interstudy heterogeneity (*I*^2^ = 2%), according to the DerSimonian-Laird FE model, revealed that VLEDs and surgery have similar effects on weight loss, and there is no significant difference between them (MD = −0.3; 95%CI = −0.74, 0.17; *P* = 0.7, >0.05) (Figure 11).

**Figure 12:**
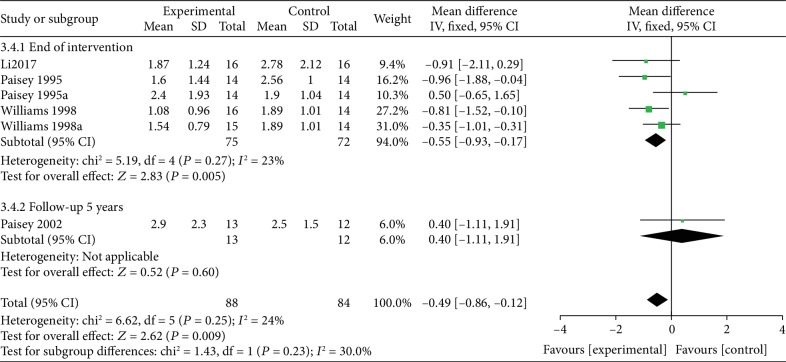
Forest plot on the mean difference in TG levels between VLED and MER controls.

*(3) VLEDs versus MER*. Four studies [37–40] analyzed the TG levels between the VLED (*n* = 88) and MER groups (*n* = 84). Results from subgroup analyses showed that a VLED was significantly better than MER in lowering TG levels (MD = −0.55; 95%CI = −0.93, −0.17; *P* = 0.005, <0.05) at the end of the intervention, with no evidence of interstudy heterogeneity (*I*^2^ = 23%; *P*_heterogeneity_ = 0.25). However, at the 5-year follow-up, similar to the result of the “Paisey et al. [37]” study, the difference in lowering TG level compared with controls was not significant (MD = 0.4; 95%CI = −1.11, 1.91; *P* = 0.60) (Figure 12).

#### 3.3.4. HOMA-IR

**Figure 13:**
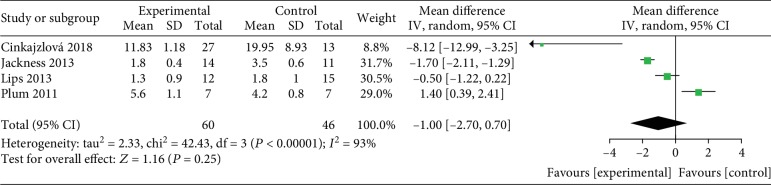
Forest plot on the mean difference in HOMA-IR between VLED and bariatric surgery controls.

Four studies [24, 32–34] analyzed the change in HOMA-IR between the VLED (*n* = 60) and bariatric surgery groups (*n* = 46), and one study analyzed the change in HOMA-IR between the VLED (*n* = 75) and MER groups (*n* = 76). The meta-analysis showed that there was no significant difference between VLEDs and surgery in increasing HOMA-IR (MD = −1; 95%CI = −2.7, 0.7; *P* = 0.25, >0.05) (Figure 13). Additionally, one study (Li et al. [39]) reported that nonsignificant improvements in HOMA-IR were also observed between the VLED and MER groups.

#### 3.3.5. Dropout

Comparing the VLED and bariatric surgery groups, no loss of patients was reported. However, most studies on VLEDs compared with those on LEDs or MER reported increased dropout rate.

**Figure 14:**
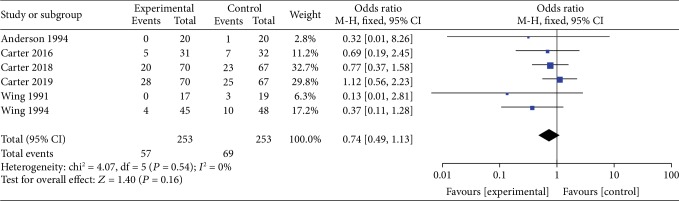
Forest plot of dropout rates between VLED and LED controls.

*(1) VLEDs versus LEDs*. Six studies [25–28, 30, 31] reported the difference in dropout rate between the VLED (*n* = 253) and LED groups (*n* = 253). The meta-analyses showed that the VLED group had a similar dropout rate with the comparator arms (OR = 0.74; 95%CI = 0.49, 1.13; *P* = 0.16, >0.05; *I*^2^ = 0%) (Figure 14).

**Figure 15:**
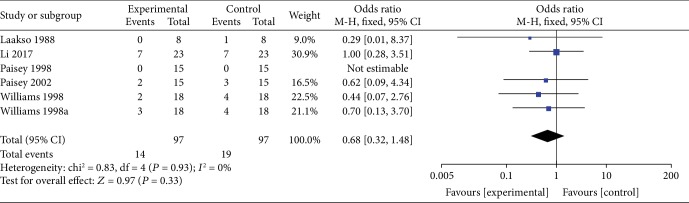
Forest plot of dropout rate between VLED and MER controls.

*(2) VLEDs versus MER*. Five studies [36, 37, 39–41] reported the difference in dropout rates between the VLED (*n* = 97) and MER groups (*n* = 97). Results from the meta-analyses showed that the VLED group had a similar dropout rate with the MER group (OR = 0.68; 95%CI = 0.32, 1.48; *P* = 0.33, >0.05) with no evidence of interstudy heterogeneity (*I*^2^ = 0%; *P*_heterogeneity_ = 0.93) (Figure 15).

#### 3.3.6. Side Effects

Nine of 18 studies involved reports of adverse reactions. Adverse reactions reported by Carter et al. [26, 27] were mainly hypoglycemia, hyperglycemia, and headache. Paisey et al. [36–38] reported adverse reactions such as hypoglycemia. myocardial infarction, and telogen effluvium. Wing et al. [30, 31] mainly reported adverse reactions such as cold intolerance, constipation, and hair loss. Andorson's study showed that frequently reported side effects during the weight loss phase included constipation, diarrhea, dizziness, and fatigue. The adverse reactions reported in Li et al.'s study were slight headache and dizziness during energy restriction. None of these studies reported significant differences in side effects between the VLED and control groups (see Table 2 for details).

#### 3.3.7. Rebound

Only three studies mentioned a rebound in body weight, blood glucose level, and other indicators after energy restriction therapy. One study [28] reported that at 24 months, in the completer analysis of 84 participants at follow-up, 44 (52%) regained weight (>1 kg weight gain) and participants regained 33% of their weight losses between 12 and 24 months. In this follow-up study, HbA1c level had increased by 0.3% (3.3 mmol/mol) from baseline at 24 months. Paisey et al. [37] found that weight loss was slower in the intensive conventional diet group than in the VLED group but better maintained at 5 years: group 1, 4.8 ± 6 kg, and group 2, 8.9 ± 4 kg. Wing et al. [30, 31] reported that, although initial weight losses were greater in the VLED group, these participants regained significantly more weight than those in the behavioural therapy group in 1 year of follow-up. Moreover, at one-year assessment, the measures of glycemic control had returned to baseline, and no differences were observed between treatment groups.

### 3.4. Publication Bias

All outcome indicators were analyzed in <10 studies, so publication bias was not examined.

### 3.5. GRADE for the Outcomes

We evaluated all outcome indicators by GRADEprofiler 3.6 from the following aspects: (1) downgrade quality of evidence, risk of bias, inconsistency, indirectness, imprecision, and publication bias and (2) upgrade quality of evidence, large effect, plausible confounding changing the effect, and dose-response gradient.

After a comprehensive analysis, the evidentiary body was formed and found that all outcome indicators had low quality or extremely low quality (see Tables 3–5 for details).

## 4. Discussion

Our systematic review provides evidence based on current clinical trials on the efficacy of continuous and intermittent VLEDs in overweight and obese individuals with T2DM by comparison to other methods of energy restriction. First, during the intervention period, a VLED is superior in the reduction of body weight and blood glucose and TG levels to LEDs and MER. After long-term follow-up, there is no obvious difference in weight loss between VLEDs and LEDs, but glycemic control is still more effective in VLEDs. Second, VLEDs offer beneficial effects on weight loss, glycemic control, and improvement of insulin resistance comparable to bariatric surgery.

Increasing evidence suggested that modest and sustained weight loss improved glycemic control in overweight and obese individuals with T2DM [3]. Furthermore, recent studies reported that intentional weight losses by low-calorie diets, usually >15 kg, could reverse T2DM into a nondiabetic state [5, 42]. Based on the current studies, our study concluded that more extreme dietary energy restriction with VLEDs is an effective method to achieve intensive weight loss in a short term and improve glycemic control more effectively compared with LEDs and MER. This conclusion supported the recommendation of the American Diabetes Association (ADA) *Standards of Medical Care in Diabetes* that high-intensity diet intervention, physical activity, and behavioural therapies to achieve a 500–750 kcal/day energy deficit and maintain >5% weight loss should be prescribed for patients with type 2 diabetes who are overweight or obese and ready to achieve weight loss. Furthermore, previous studies showed that rapid weight loss by VLEDs is inevitably followed by weight regain [42], but recent studies with at least 1-year follow-up found that VLEDs might present a longer effect on weight maintenance [5, 43]. Another study showed that even though weight was regained, the short-term weight loss had long-lasting benefits on glycemic control and prevention of cardiovascular effects in T2DM [44]. Our results are in line with this study. After further analysis of the effects of long-term follow-up (1–5 years), we found no obvious difference in weight loss between VLEDs and LEDs, but VLEDs still maintained better glycemic control. The lasting effects of VLEDs may be attributed to improved insulin sensitivity remaining from weight loss [45], “metabolic memory” from the treatment period [46], and “legacy effect” by lifestyle intervention [47].

It is reported that dyslipidemia, especially hypertriglyceridemia, is an independent risk factor in predicting the development of diabetes, which is partially mediated by insulin resistance and obesity [48]. Several prospective studies have demonstrated that weight loss induced decreases in pancreatic and liver TG levels in T2DM, which was associated with the recovery of insulin secretory function [6, 49]. However, the effect of weight loss by VLEDs on the plasma TG level is rarely discussed. Our meta-analyses found that VLEDs reduced the plasma TG level in T2DM more effectively compared to LEDs and MER and had an equivalent effect with bariatric surgery, which may have potential effect on preventing the development of T2DM.

Bariatric surgery is confirmed to have superior effect in T2DM [50] and has been proposed as a first-line therapy for obese patients with T2DM [3]. Bariatric surgery can restore normal liver insulin sensitivity within days and decrease plasma glucose and TG levels within weeks [51]. In this context, some studies determined whether the effects of bariatric surgery are primarily due to negative energy balance or unique to the surgical procedure [24, 51]. Our study shows that VLEDs are as effective as bariatric surgery (mainly RYGB) in terms of weight loss, glycemic control, insulin resistance improvement, and plasma TG level reduction. Additionally, VLEDs have lower costs and lesser adverse effects compared with bariatric surgery. Thus, VLEDs may be a considerable therapy when patients could not or would not wish to undergo surgical treatments.

VLEDs were found to be acceptable as indicated by the low dropout rate in both this and a previous study. The main reason may be that rapid weight loss increases patient's confidence, and hunger of patients after VLED intervention is more inhibited. A study shows that attrition was lower when weight loss was undertaken rapidly rather than gradually, because rapid weight loss might motivate participants [52]. Moreover, ketosis suppresses appetite and increases the satiety hormone cholecystokinin, which increases the possibility that participants with rapid weight loss might have been less hungry during the weight loss phase than those following the gradual diet [53–56]. Of note, the experience of healthcare professionals involved in the trial in obesity treatment also had a significant impact on attrition.

While the short-term efficacy of VLEDs is evident and patient compliance is acceptable according to our analysis, the reports of adverse reactions in the studies are incomplete, limiting the use of this method.

In a previous systematic review, Rehackova et al. [19] revealed that VLEDs led to considerable weight loss and blood glucose control via small sample or qualitative studies. However, evidence on the long-term efficacy of VLEDs with regard to weight loss in individuals with T2DM is lacking. Our study has expanded the sample size and further analyzed the follow-up results between VLEDs and LEDs. After the follow-up (1–5 years), VLEDs present a more effective glycemic control effect, but there is no obvious difference in weight loss between VLEDs and LEDs. Some included studies [28, 30, 31, 37] also showed that, at the end of VLED intervention, the decrease in body weight, blood glucose level, and other indicators would rebound to varying degrees. This shows that adherence to a VLED regimen is crucial in maximizing intervention effects. It has been shown that greater initial weight loss facilitates weight maintenance if followed by an effective weight loss maintenance programme [57]. Further exploring a strategy to suppress hunger after rapid weight loss and prevent weight regain of VLEDs is greatly important in the popularization of this method.

This meta-analysis provides some objective evidence for the application of VLEDs in obese individuals with T2DM, but there are still many limitations in the study. First, both non-RCTs and RCTs were combined in the meta-analyses, which increased the heterogeneity and risk of bias. Therefore, the results of this study still need to be confirmed by higher-quality research. Second, some high-quality research in this field has been conducted by a small number of research groups, resulting in insufficient representation of data. Thus, more extensive studies are needed to clarify the practicability of VLEDs in different ethnic groups. Third, most included studies did not mention the use of hypoglycemic drugs in participants. When VLEDs are used to intervene with obese patients with T2DM, determination of hypoglycemic drugs is difficult. In the future, the standardized research of this area should be strengthened. Lastly, only a few included studies that recorded follow-up results, which led to insufficient convincing evidence. Moreover, the longest follow-up duration in the included studies was only 5 years, so the long-term effect of VLEDs needs further study.

## 5. Conclusions

Dietary intervention through VLEDs is more effective in rapid weight loss and glycemic control and improved lipid metabolism in overweight and obese individuals with T2DM than LEDs and MER, although they have similar long-term effects. Moreover, VLEDs have similar efficacy and acceptability with bariatric surgery, which shows that VLEDs have considerable curative effect for remission of T2DM. However, after GRADE, it was found that all outcome indicators had low quality or base quality, so the results of this study still need to be further confirmed by high-quality research.

## Figures and Tables

**Table 1:** Characteristics of included studies.

Study ID	Study type	Interventions (kcal/day)	Comparator (kcal/day)	Use of hypoglycemic drugs		Study duration	
				VLED	Control	Treatment	Follow-up
Anderson 1994	RCT	VLED (800): at least five liquid supplements/day which provide 800 kcal with 80 g of high-quality protein+two vitamin/mineral tablets	LED (approximately 820): at least three supplements/day which provide 320 kcal and 32 g of high-quality protein, one vitamin/mineral tablet, recommended evening meal of approximately 500 kcal and 50 g of high-quality protein	Unclear	Unclear	3 months	1 year

Carter 2016	RCT	Intermittent VLED (400-598): 1670-2500 kJ/day for 2 days each week, with the remaining 5 days as habitual eating	LED (1196-1555): continuous energy restriction diet of 5000-6500 kJ/day	Medications adjusted according to blood glucose level		12 weeks	None

Carter 2018	RCT	VLED (500-600): an intermittent energy restriction diet (500-600 kcal/d) followed for 2 nonconsecutive days per week (participants followed their usual diet for the other 5 days)	LED (1200-1500): a continuous energy restriction diet (1200-1500 kcal/d) followed for 7 days per week	Medications could be reduced depending on glucose		12 months	2 years

Carter 2019	RCT	VLED (500-600): an intermittent energy restriction diet (500-600 kcal/d) followed for 2 nonconsecutive days per week (participants followed their usual diet for the other 5 days)	LED (1200-1500): a continuous energy restriction diet (1200-1500 kcal/d) followed for 7 days per week	Medications could be reduced depending on glucose		12 months	2 years

Harvey 1993	RCT	VLED (400-500): during weeks 1-12, consumed a 400-500 kcal/day. This was followed by a 6-week refeeding period, which required slow reintroduction of calories, carbohydrate, and fat. By the end of the 6 weeks of refeeding, subjects in the VLED were prescribed a self-selected balanced, low-calorie diet (1000-1200 kcal/day)	LED (1000-1200): a self-selected balanced diet of 1000-1200 kcal per day for 6 months	15 of the subjects were treated with diet only, 54 with oral medication, and 23 with insulin.		24 week	None

Wing 1991	RCT	VLED (400-500): weeks 0–4: 1000–1500 kcal/day; weeks 5–12: 400–500 kcal/day; weeks 13–20: 1000 kcal/day; weeks 21–72 : 1000–1500 kcal/day (weight maintenance). Included a 20-week behavioural treatment programme with weekly group meetings including instructions on behavioural modification, exercise, and diet	LED (1000–1500): weeks 0-20: 1000–1500 kcal/day (intervention period); weeks 21-72: 1000–1500 kcal/day (weight maintenance). Included a 20-week behavioural treatment programme with weekly group meetings including instructions on behavioural modification, exercise, and diet	Unclear	Unclear	20 weeks	1 year

Wing 1994	RCT	VLED (400-500): weeks 0–12 and 24-36: 400–500 kcal/d+vitamins and supplements, otherwise 1000-1200 kcal/d. Included a 50-week behavioural treatment programme with weekly group meetings including instructions on behavioural modification, exercise, and diet	LED (1000-1200): weeks 0-48: 1000-1200 kcal/d (intervention period); subjects were encouraged to keep their fat intake below 30% of the daily calorie intake. Included a 50-week behavioural treatment programme with weekly group meetings including instructions on behavioural modification, exercise, and diet	Unclear	Unclear	1 year	2 years

Jackness 2013	NRCT	VLED (300-500): day 1: 360 kcal/day; days 2–24: 500 kcal/day	RYGB (500): postoperative VLED is assumed of approximately 500 kcal/day until the end of week 3	Unclear	Unclear	Mean study period of 21 days	None

Lips 2013	NRCT	VLED: weeks 0–3: 600 kcal/day (intervention period); weeks 3–8: 800–1000 kcal/day; after 2 months: 1200 kcal/day	RYGB (<800): first 5 days after RYGB operation: <600 kcal/day; weeks 1–3: gradual increase to 700–800 kcal/day; week 3–month 3: 1200 kcal/day	Unclear	Unclear	3 months	None

Plum 2011	NRCT	VLED (800): the diet divided into five servings of 160 kcal (800 kcal/day) in 237 ml per serving	RYGB: postintervention, subjects followed dietary instructions provided by the surgical team	55% reduction in the number of medications after LCD	Antidiabetic medications were discontinued after RYGB	VLED: 8.1 (0.5) weeks RYGB: 3.4 (0.3) weeks	None

Cinkajzlova 2018	NRCT	VLED (595): total energy content of 2500 kJ/day for 3 weeks	Surgery: the procedures included gastric plication (10 subjects), gastric banding (2 subjects), and gastric bypass (1 subject)	Unclear	Unclear	VLCD 3 weeks; control 1 m, 3 m, and 1 y	None

Steven 2016	RCT	VLED (700): the VLED provided an average of 700 kcal/day	RYGB (~800): the postoperative (RYGB) diet was water only on day 1 then a semisolid diet (~800 kcal/day) for the rest of the first week.	Participants were asked to stop medications prior to the first study: metformin and/or sulphonylureas for at least 72 h, dipeptidyl peptidase-4 inhibitors for 1 month, and insulin for at least 24 h		7 days	None

Paisey 1995	NRCT	VLED (400-670): 400-470 kcal/d for women, 540–670 kcal/d for men for 3-5 months and repeated in the course of the study if appropriate. Once an agreed target weight had been reached, patients were seen intensively to wean them back onto a low-fat diet. A standardized programme of low-fat, low-refined carbohydrate foods was introduced over a 6-week period as patients transferred from VLED to normal eating patterns. They were advised to continue low-fat nutrition in the long term with three main meals daily	MER: low-fat, low-sugar, and high-fibre intake advised; 5-day self-report food records were collected and discussed individually, repeated every 6-8 weeks. Aerobic exercises with encouragement performed at each visit followed by a group discussion on nutrition	All antidiabetic medication was stopped on day one, and insulin dosage halved. Hypotensive and hypolipidemic agents were stopped if appropriate at one month	Unclear	6 months	None

Paisey 1998	NRCT	Same as “Paisey 1995”	Same as “Paisey 1995”	Subjects were advised to stop all antidiabetic medication and diuretics from day 1 of treatment	Unclear	12 months	None

Paisey 2002	NRCT	Same as “Paisey 1995”	Same as “Paisey 1995”	Antidiabetic and antihypertensive medications were stopped during the first week of treatment	Unclear	At least 6 weeks	5 years

Li 2017	RCT	VLED (300): days 1~2: low-calorie diet (1200 kcal/day); from the evening of study day 3 to the evening of study day 11: 300 kcal/day; followed by 3 low-calorie diet days (1200 kcal/day), followed by advice about a Mediterranean diet. Fasting took place only once in the 4-month period	MER: follow the principles of a Mediterranean diet	Subjects were advised to abstain from other new treatments against diabetes during the study period		4 months	None

Williams 1998	RCT	VLED (400-600): 1500-1800 kcal/day diet, except for a total of 20 study days during which they consumed a 400-600 kcal/day VLED. (1-day): a VLED for 5 consecutive days during week 2 of the study and then 1 day a week for 15 weeks, subjects used diaries to record daily caloric intake	MER (1500-1800): a 1500-1800 kcal/day diet throughout the 20 weeks of the treatment programme. Included a 20-week behavioural treatment programme with weekly group meetings including instructions on behavioural modification, exercise, and diet. Subjects used diaries to record daily caloric intake	Unclear	Unclear	20 weeks	None

Williams 1998a	RCT	VLED (400-600): 1500-1800 kcal/day diet, except for a total of 20 study days during which they consumed a 400-600 kcal/day VLED. (5-day): a VLED for 5 consecutive days during weeks 2, 7, 12, and 17+a 20-week behavioural treatment programme with weekly group meetings including instructions on behavioural modification, exercise, and diet. Subjects used diaries to record daily caloric intake	MER (1500-1800): a 1500-1800 kcal/day diet throughout the 20 weeks of the treatment programme. Included a 20-week behavioural treatment programme with weekly group meetings including instructions on behavioural modification, exercise, and diet. Subjects used diaries to record daily caloric intake	Unclear	Unclear	20 weeks	None

Laakso 1988	RCT	VLED (500-800): day 1-day 3: 30 kcal/kg/d; day 4-day 15: 500 kcal/d; day 15-day 17: 800 kcal/d	MER (30 kcal/kg/d): the diet was as previously described (30 kcal/kg/d) consisting of 50% carbohydrates, 30% fat, and 20% protein divided into three main meals	All medications for diabetes were discontinued	Insulin was started using intermediate-acting insulin as one single injection at 7 AM. The mean dosage of insulin (±SEM) was 39 ± 5 U/d	2 weeks	None

VLED: very low-energy diet; LED: low-energy diet; MER: mild energy restriction; RYGB: Roux-en-Y gastric bypass; RCT: randomized controlled trial; NRCT: nonrandomized controlled trial.

**Table 2:** Side effects.

Study ID	VLED	Control
Carter 2016	Hypoglycemia (<4 mmol/l) only occurred in insulin-controlled participants (*n* = 6), with no difference between treatment groups	
Carter 2018	Hypoglycemia (*n* = 2) Hyperglycemia (*n* = 3) Headache (*n* = 2)	Hypoglycemia (*n* = 6) Hyperglycemia (*n* = 7)
Paisey 1995	Severe hypoglycemic attack (*n* = 1)	Myocardial infarction (*n* = 1)
Paisey 1998	Nonfatal myocardial infarction (*n* = 1) Severe hypoglycemic attack (*n* = 1)	Nonfatal myocardial infarction (*n* = 1)
Paisey 2002	Nonfatal myocardial infarction (*n* = 1) Telogen effluvium (*n* = 6, which recovered within 2 years of stopping VLEDs in five)	Primary biliary cirrhosis (*n* = 1) Nonfatal myocardial infarction (*n* = 1)
Wing 1991	Coldness, constipation, dry skin, diarrhea, dizziness, vomiting, or weakness—commonly reported side effects of VLEDs. There were no significant differences over time in any of these symptoms and no significant difference between subjects in the LED and VLED groups. However, uric acid increased significantly in the VLED group	
Wing 1994	Common side effects included cold intolerance, constipation, and hair loss, which all resolved when the VLED was terminated	Unclear
Andorson 1994	Frequently reported side effects during the weight loss phase included constipation (56% of subjects), diarrhea (31%), dizziness (31%), fatigue (31%), flu/sore throat (13%), headache (10%), vomiting (10%), blurred vision (10%), muscle cramps (8%), and syncope (5%). None of these side effects required treatment alteration.	
Li 2017	No serious adverse effects: slight headache (*n* = 3); slight dizziness (*n* = 1)	No serious adverse effects

VLED: very low-energy diet; LED: low-energy diet.

**Table 3:** The GRADE evidence of VLEDs compared to LEDs for overweight and obese people with type 2 diabetes mellitus.

Quality assessment							No. of patients		Effect		Quality	Importance
No. of studies	Design	Risk of bias	Inconsistency	Indirectness	Imprecision	Other considerations	VLED	LED	Relative (95% CI)	Absolute		
Weight (better indicated by lower values)												
8	Randomized trials	Serious^1^	No serious inconsistency	Serious^2^	No serious imprecision	None	246	241	—	MD -1.86 lower (-3.34 to -0.37 lower)	Low	9
Weight: end of the intervention (better indicated by lower values)												
5	Randomized trials	Serious^1^	No serious inconsistency	Serious^2^	No serious imprecision	None	151	146	—	MD -2.77 lower (-4.81 to -0.72 lower)	Low	9
Weight: follow − up ≥ 1 year (better indicated by lower values)												
3	Randomized trials	Serious^1^	No serious inconsistency	Serious^2^	Serious^3^	None	95	95	—	MD -0.84 lower (-3.01 lower to 1.32 higher)	Very low	9
Glucose (better indicated by lower values)												
6	Randomized trials	Serious^1^	No serious inconsistency	Serious^2^	No serious imprecision	None	187	180	—	MD -1.26 lower (-1.97 to -0.55 lower)	Low	8
Glucose: end of the intervention (better indicated by lower values)												
3	Randomized trials	Serious^1^	No serious inconsistency	Serious^2^	No serious imprecision	None	75	76	—	MD -1.18 lower (-2.05 to -0.3 lower)	Low	8
Glucose: follow − up ≥ 1 year (better indicated by lower values)												
3	Randomized trials	Serious^1^	No serious inconsistency	Serious^2^	Serious^4^	None	112	104	—	MD -1.43 lower (-2.65 to -0.2 lower)	Very low	8
TG (better indicated by lower values)												
6	Randomized trials	Serious^1^	No serious inconsistency	Serious^2^	No serious imprecision	None	185	179	—	MD 0.31 lower (-0.5 to -0.13 lower)	Low	7
TG: end of the intervention (better indicated by lower values)												
3	Randomized trials	Serious^1^	No serious inconsistency	Serious^2^	No serious imprecision	None	75	76	—	MD -0.35 lower (-0.58 to -0.12 lower)	Low	7
TG: follow − up ≥ 1 year (better indicated by lower values)												
3	Randomized trials	Serious^1^	No serious inconsistency	Serious^2^	Serious^4^	None	110	103	—	MD -0.25 lower (-0.55 lower to 0.06 higher)	Very low	7
Dropout												
6	Randomized trials	Serious^1^	No serious inconsistency	Serious^2^	No serious imprecision	None	57/253 (22.5%)	69/253 (27.3%)	OR 0.74 (0.49 to 1.13)	56 fewer per 1000 (from 118 fewer to 25 more)	Low	6
								21.4%		46 fewer per 1000 (from 96 fewer to 21 more)		

^∗^CI: confidence interval; OR: odds ratio. GRADE Working Group grades of evidence: high quality: further research is very unlikely to change our confidence in the estimate of effect; moderate quality: further research is likely to have an important impact on our confidence in the estimate of effect and may change the estimate; low quality: further research is very likely to have an important impact on our confidence in the estimate of effect and is likely to change the estimate; very low quality: we are very uncertain about the estimate. ^1^There are studies that do not account for specific stochastic methods, so they are reduced by one level. ^2^Interventions include continuous VLEDs and intermittent VLEDs, which differ to some extent, so they are reduced by one level. ^3^No explanation was provided. ^4^The ratio of 95% CI to the effect is more than 50%. 95% CI is wider and its accuracy is poor, so it decreases one level.

**Table 4:** The GRADE evidence of VLEDs compared to bariatric surgery for overweight and obese people with type 2 diabetes mellitus.

Quality assessment							No. of patients		Effect		Quality	Importance
No. of studies	Design	Risk of bias	Inconsistency	Indirectness	Imprecision	Other considerations	VLED	Bariatric surgery	Relative (95% CI)	Absolute		
Weight												
4	Randomized trials	Serious^1^	No serious inconsistency	No serious indirectness	Serious^2^	None	42	42	—	MD -3.14 lower (-10.04 lower to 3.67 higher)	Low	9
Glucose (better indicated by lower values)												
5	Randomized trials	Serious^1^	No serious inconsistency	No serious indirectness	Serious^2^	None	69	55	—	MD 0.37 higher (-0.22 lower to 0.96 higher)	Low	8
TG (better indicated by lower values)												
4	Randomized trials	Serious^1^	No serious inconsistency	No serious indirectness	Serious^2^	None	57	40	—	MD -0.04 lower (-0.25 lower to 0.17 higher)	Low	7
HOMA-IR (better indicated by lower values)												
4	Observational studies^3^	No serious risk of bias	Serious^4^	No serious indirectness	Serious^2^	None	60		—	—	Very low	6
								46		MD -1 lower (-2.7 lower to 0.7 higher)		

^∗∗^CI: confidence interval; OR: odds ratio. GRADE Working Group grades of evidence: high quality: further research is very unlikely to change our confidence in the estimate of effect; moderate quality: further research is likely to have an important impact on our confidence in the estimate of effect and may change the estimate; low quality: further research is very likely to have an important impact on our confidence in the estimate of effect and is likely to change the estimate; very low quality: we are very uncertain about the estimate. ^1^Some studies were nonrandomized controlled trials. ^2^The ratio of 95% CI to the effect is more than 50%. 95% CI is wider and its accuracy is poor, so it decreases one level. ^3^Case-control. ^4^The *I*^2^ value of the combined results is larger, and there is statistical heterogeneity, so it falls by one grade.

**Table 5:** The GRADE evidence of VLEDs compared to mild energy restriction for overweight and obese people with type 2 diabetes mellitus.

Quality assessment							No. of patients		Effect		Quality	Importance
No. of studies	Design	Risk of bias	Inconsistency	Indirectness	Imprecision	Other considerations	VLED	Mild energy restriction	Relative (95% CI)	Absolute		
Weight (better indicated by lower values)												
6	Randomized trials	Very serious^1,2^	Serious^3^	Serious^4^	No serious imprecision	None	88	88	—	MD -4.57 lower (-9.44 lower to 0.3 higher)	Very low	9
Weight: end of the intervention (better indicated by lower values)												
5	Randomized trials	Very serious^1,2^	Serious^3^	Serious^4^	No serious imprecision	Strong association^5^	75	76	—	MD -6.72 lower (-10.05 to -3.39 lower)	Very low	9
Weight: follow-up 5 years (better indicated by lower values)												
1	Observational studies	Serious^6^	No serious inconsistency	No serious indirectness	Serious^7^	None	13	12	—	MD 4.1 higher (0.13 to 8.07 higher)	Very low	9
Glucose (better indicated by lower values)												
6	Randomized trials	Very serious^1,2^	No serious inconsistency	Serious^4^	No serious imprecision	None	86	84	—	MD -0.75 lower (-1.44 to -0.06 lower)	Very low	8
Glucose: end of the intervention (better indicated by lower values)												
5	Randomized trials	Very serious^1,2^	No serious inconsistency	Serious^4^	No serious imprecision	None	74	72	—	MD -0.74 lower (-1.44 to -0.04 lower)	Very low	8
Glucose: follow-up 5 years (better indicated by lower values)												
1	Observational studies	Serious^6^	No serious inconsistency	No serious indirectness	Serious^7^	None	12	12	—	MD -1 lower (-4.62 lower to 2.62 higher)	Very low	8
TG (better indicated by lower values)												
6	Randomized trials	Very serious^1,2^	No serious inconsistency	Serious^4^	No serious imprecision	None	88	84	—	MD -0.49 lower (-0.86 to -0.12 lower)	Very low	7
TG: end of the intervention (better indicated by lower values)												
5	Randomized trials	Very serious^1,2^	No serious inconsistency	Serious^4^	No serious imprecision	None	75	72	—	MD -0.55 lower (-0.93 to -0.17 lower)	Very low	7
TG: follow-up 5 years (better indicated by lower values)												
1	Observational studies^8^	Serious^6^	No serious inconsistency	No serious indirectness	No serious imprecision	None	13		—	—	Very low	7
								12		MD 0.4 higher (-1.11 lower to 1.91 higher)		
Dropout												
6	Randomized trials	Very serious^1,2^	No serious inconsistency	No serious indirectness	No serious imprecision	None	14/97 (14.4%)	19/97 (19.6%)	OR 0.68 (0.32 to 1.48)	54 fewer per 1000 (from 124 fewer to 69 more)	Low	6
								21.1%		57 fewer per 1000 (from 132 fewer to 73 more)		

^∗^CI: confidence interval; OR: odds ratio. GRADE Working Group grades of evidence: high quality: further research is very unlikely to change our confidence in the estimate of effect; moderate quality: further research is likely to have an important impact on our confidence in the estimate of effect and may change the estimate; low quality: further research is very likely to have an important impact on our confidence in the estimate of effect and is likely to change the estimate; very low quality: we are very uncertain about the estimate. ^1^Some studies were nonrandomized controlled trials. ^2^There are studies that do not account for specific stochastic methods, so they are reduced by one level. ^3^The *I*^2^ value of the combined results is larger and there is statistical heterogeneity, so it falls by one grade. ^4^Interventions include continuous VLEDs and intermittent VLEDs, which differ to some extent, so they are reduced by one level. ^5^After merger, the effect is large and the accuracy is high, so it goes up one level. ^6^Failure to adequately control confounding factors. ^7^The ratio of 95% CI to the effect is more than 50%. 95% CI is wider and its accuracy is poor, so it decreases one level. ^8^Case-control.

## Abbreviations

DM: Diabetes mellitus
VLED: Very low-energy diet
LED: Low-energy diet
MER: Mild energy restriction
RYGB: Roux-en-Y gastric bypass
CI: Confidence interval
MD: Mean difference.
